# Serum Biomarkers in Patients with Relapsing Eosinophilic Granulomatosis with Polyangiitis (Churg-Strauss)

**DOI:** 10.1371/journal.pone.0121737

**Published:** 2015-03-26

**Authors:** Christian Dejaco, Bastian Oppl, Paul Monach, David Cuthbertson, Simon Carette, Gary Hoffman, Nader Khalidi, Curry Koening, Carol Langford, Kathleen McKinnon-Maksimowicz, Philip Seo, Ulrich Specks, Steven Ytterberg, Peter A. Merkel, Jochen Zwerina

**Affiliations:** 1 Department of Rheumatology and Immunology, Medical University of Graz, Graz, Austria; 2 1^st^ Medical Department and Ludwig Boltzmann Institute of Osteology, Hanusch Hospital, Vienna, Austria; 3 Section of Rheumatology, Boston University School of Medicine, Boston, MA, United States of America; 4 Department of Pediatrics, University of South Florida College of Medicine, Tampa, FL, United States of America; 5 Division of Rheumatology, Mount Sinai Hospital, Toronto, ON, Canada; 6 Department of Rheumatology, Cleveland Clinic, Cleveland, OH, United States of America; 7 Division of Rheumatology, McMaster University, Hamilton, ON, Canada; 8 Division of Rheumatology, University of Utah, Salt Lake City, UT, United States of America; 9 George E. Wahlen Department of Veterans Affairs Medical Center, Salt Lake City, UT, United States of America; 10 Division of Rheumatology, University of Pittsburgh, Pittsburgh, PA, United States of America; 11 Division of Rheumatology, Johns Hopkins University, Baltimore, MD, United States of America; 12 Division of Pulmonary and Critical Care Medicine, Mayo Clinic College of Medicine, Rochester, MN, United States of America; 13 Division of Rheumatology, Mayo Clinic College of Medicine, Rochester, MN, United States of America; 14 Division of Rheumatology, University of Pennsylvania, Philadelphia, PA, United States of America; 15 Department of Internal Medicine 3, University of Erlangen-Nuremberg, Erlangen, Germany; Nippon Medical School Graduate School of Medicine, JAPAN

## Abstract

**Introduction:**

Previous studies suggest a role for eotaxin-3, TARC/CCL17 and IgG4 in newly- diagnosed patients with eosinophilic granulomatosis with polyangiitis (EGPA, Churg-Strauss) with highly active disease. The role of these biomarkers in relapsing disease is unclear.

**Methods:**

Serum levels of TARC/CCL17, eotaxin-3, IgG4, and IgG4/IgG ratio were determined in serum samples from a longitudinal cohort of patients with EGPA (105 visits of 25 patients). Epidemiological, clinical and laboratory data were available for all visits.

**Results:**

At the first visit, 80% of patients were using glucocorticoids and 68% additional immunosuppressive drugs. Disease flares were seen at 18 visits. The median BVAS and BVAS/WG scores at time of relapse were 4 and 2, respectively. None of the biomarkers tested were useful to discriminate between active disease and remission. Patients treated with prednisone had lower eotaxin-3 and eosinophil levels compared to patients not taking glucocorticoids irrespective of disease activity. Use of immunosuppressive agents was not associated with biomarker levels.

**Conclusions:**

Serum levels of TARC/CCL17, eotaxin-3, IgG4, and IgG4/IgG ratio do not clearly differentiate active and inactive disease in established EGPA. Defining biomarkers in EGPA remains a challenge especially during times of glucocorticoid use.

## Introduction

Eosinophilic granulomatosis with polyangiitis (EGPA, Churg-Strauss) is a rare systemic vasculitis almost exclusively arising in asthma patients [1]. Although EGPA can occur in any organ system, the most frequent disease manifestations are confined to the respiratory tract and peripheral nervous system. Skin, gastro-intestinal tract, and cardiac involvement are also common, with the latter being the major determinant of mortality in the long- term [2]. Marked eosinophilia is an extremely common feature of EGPA. Anti-neutrophil cytoplasmic antibodies (ANCA) are present in one third of patients at diagnosis and are associated with the presence of glomerulonephritis [3]. On a microscopic level, EGPA typically causes an eosinophil- rich, granulomatous, necrotizing vasculitis [4]. Mild disease is usually treated with glucocorticoids alone, whereas severe disease requires additional therapy with immunosuppressive drugs such as cyclophosphamide [5,6].

Relapses are common in EGPA and clinical experience suggests that severe flares are usually accompanied by blood eosinophilia [7]. However, intermittent elevation of eosinophil count without change in clinical activity of EGPA itself is also frequent. Moreover, discriminating disease activity from worsening of underlying asthma and sinusitis is challenging. Thus, biomarkers supporting the diagnosis of disease flares in EGPA would be extremely helpful. In prior studies of untreated patients with EGPA with active disease, we found several potential biomarkers of disease activity including serum levels of eotaxin-3, TARC/CCL17, and IgG4 [8–10]. In the current study, we investigated a panel of biomarkers in a prospective, longitudinal EGPA cohort to determine the marker’s value in identifying relapsing disease.

## Methods

### Study patients

Twenty-five patients enrolled between 2006 and 2012 in the Vasculitis Clinical Research Consortium (VCRC) Longitudinal Study of EGPA were selected for this analysis. The VCRC is a multicenter research infrastructure supported by US National Institutes of Health (NIH). Patients fulfilled the 1990 American College of Rheumatology (ACR) classification criteria for Churg-Strauss syndrome [11]. All subjects were enrolled using protocols and written informed consent documents approved by local institutional review boards/ethics committees at all institutions at which participants were recruited: Boston University (Boston University Medical Campus IRB), University of South Florida (USF IRB), McMaster University (McMaster Research Ethics Board), Mount Sinai Hospital Toronto (The Mount Sinai Research Ethics Board), Cleveland Clinic (IRB Cleveland Clinic Foundation), University of Utah (University of Utah IRB), University of Pittsburgh (University of Pitts burgh IRB), Johns Hopkins University (The Johns Hopkins Medicine IRB), Mayo Clinic (Mayo Clinic Institutional Review Board) and University of Pennsylvania (University of Pennsylvania IRB).

Clinicians with expertise in vasculitis performed comprehensive clinical assessments at each visit. Features of disease directly attributable to EGPA were recorded using standardized data collection forms. Summary measures of disease activity at each visit included calculation of the Birmingham Vasculitis Activity Score (BVAS) and the BVAS for Wegener’s Granulomatosis (BVAS/WG) [12,13]. BVAS and BVAS/WG scores indicate new or worse disease activity within the prior four weeks. In addition, physician-investigators categorically rated patients’ disease activity within the past four weeks as either remission, mild, moderate or high disease activity. For statistical analyses, we pooled data from patients’ visits with mild, moderate and high disease activity into one category named “active”. All patients’ visits categorized as active revealed by BVAS/WG and BVAS. Physician global assessment of disease activity within the prior 28 days was evaluated on a 0–10 Likert scale (BVAS/WG PGA). Worsening asthma alone in the absence of involvement of other organ systems was not sufficient to classify a patient as active EGPA.

All 18 patients with EGPA in the VCRC cohort (as of 2012) who had a clinical relapse and who had additional samples taken during remission were investigated. Serum samples were retrieved at the relapse visits, and further sampling (1–7 per patient) was performed at visits conducted in phases of remission 1–8 months before and/or 3–24 months after a flare. Seven patients with EGPA without clinical relapse were studied in a similar way: 2–5 serum samples across 3–12 months.

### Biomarker studies

Results of the following tests were obtained for all patients at all study visits: Erythrocyte sedimentation rate (ESR, mm/hour), C-reactive protein (CRP, mg/L), and eosinophil count were measured at the VCRC sites using standard methods. Serum levels of eotaxin-3 and TARC/CCL17 were determined by ELISA (both R&D Systems, Minneapolis, MN, USA) as previously described [8,10]. Serum levels of total IgG and IgG4 were analyzed by nephelometry.

### Statistical analysis

Descriptive statistics were used to summarize data. The Kolmogorov-Smirnov test was used to check for normality. For data with a normal distribution, mean ± standard deviation is presented whereas for non-normally distributed data the median and range is reported. We used the Mann-Whitney U test for comparisons of (not normally distributed) data. Proportions were analyzed by chi-square or Fisher’s exact test, as appropriate. Statistical analysis was performed using SPSS version 21 (SPSS Inc., Chicago, IL).

Multivariate inclusive logistic regression (maximum likelihood method) models were conducted to investigate the association between biomarkers, disease activity and glucocorticoid use. There was insufficient power to perform a repeated measurement model due to the low number of patients and visits. Therefore, we analyzed each visit as if it was an independent case and adjusted for possible clustering of findings within patients including the factor patient into the regression model. We applied either disease activity (active or remission as determined by treating physicians), the use of glucocorticoids (yes or no) or treatment with immunosuppressive agents (yes or no) as variables of primary interest. Glucocorticoids, immunosuppressive agents and disease activity were included as covariates in these models. Goodness of fit was tested with Hosmer-Lemeshow statistics and for sensitivity analysis we excluded cases producing low/high DFBETAs and/or large Cook values.

## Results

### Patient Characteristics

We analyzed 105 visits of 25 patients with EGPA (median 4 visits per patient, range 2–8) (**Table 1**). Mean age of the patients was 49.7 ± 13 years with similar numbers of males and females. At their first visits, 20 patients (80%) were receiving prednisone and 17 (68%) had additional immunosuppressive therapies (9 methotrexate, 7 azathioprine and 1 mycophenolate mofetil). Remission was observed in 87 (83%) visits, while disease flares were seen at 18 (17%) visits. Five of the 18 flares observed were characterized as moderate or severe, while the other 13 were regarded as mild. No patient had more than one flare. Median BVAS at flare visits was 4 (range 0–18) and median BVAS/WG was 2 (range 1–4). EGPA symptoms most commonly observed in relapsing patients were: constitutional symptoms, musculoskeletal pain, sinusitis and peripheral neuropathy. Cardiovascular events, renal and gastrointestinal disease manifestations were rare or absent during relapsing disease in this cohort.

**Table 1:** Clinical manifestations of study patients with EGPA.

Clinical characteristics	Remission, n = 87	Active, n = 18
Constitutional symptoms, n (%)	1 (1.2)	6 (33.3)***
Arthralgia/Arthritis, n (%)	0	4 (22.2) ***
Myalgia/Myositis, n (%)	0	1 (5.6)
Skin, n (%)	2 (2.3)	2 (11.2)
Ear nose throat, n (%)	2 (2.3)	5 (27.8)**
Cardiovascular Events, n (%)	0	1 (5.6)
Gastrointestinal, n (%)	1 (1.2)	0
Pulmonary, n (%)	2 (2.3)	2 (11.2)
Asthma, n (%)	19 (21.8)	3 (16.7)
Kidney		
Proteinuria, n (%)	7 (8.0)	3 (16.7)
Hematuria, n (%)	6 (6.9)	2 (11.1)
RBC casts, n (%)	3 (3.4)	1 (5.6)
Elevated serum creatinine, n (%)	3 (3.4)	1 (5.6)
Creatinine, median (range) [mg/dL]	0.8 (0–1.6)	0.8 (0.6–1.7)
Peripheral neuropathy, n (%)	2 (2.3)	3 (16.7)*
BVAS/WG PGA, median (range)	0 (0)	2.5 (1–8) ‡
BVAS score, median (range)	0	4 (0–18) ‡
BVAS/WG score, median (range)	0	2 (1–4) ‡
IgE, median (range)	16.0 (0.1–1926.0)	0 (0.1–427.0)
WBC, median (range)	7.3 (3.2–13.9)	8.8 (4.8–12.8) †

EGPA: eosinophilic granulomatosis with polyangiitis (Churg-Strauss); BVAS: Birmingham Vasculitis Activity Score; BVAS/WG: BVAS for Wegener’s Granulomatosis; n (%), number and percentage of patients (out of those with known outcome) with manifestation since previous visit

*p<0.05

**p<0.01

***p<0.001 according to Fisher’s exact test (not corrected for multiple testing).

† p<0.05

‡ p<0.001 according to Mann Whitney U test (not corrected for multiple testing)

### Serum levels of eotaxin-3, TARC/CCL17 and IgG4 in patients with EGPA

Serum levels of eotaxin-3, TARC/CCL17, IgG4, and IgG4/IgG ratio did not discriminate between periods of remission and active disease within individual patients (**Table 2**), even after adjustment for prednisone use. Similarly, ESR, CRP, and eosinophil count also failed to differentiate between active and inactive disease.

**Table 2:** Serum biomarkers of patients with EGPA in remission and at relapse.

Serum parameter	Remission (n = 87)	Active (n = 18)	MWU	LR
Eotaxin-3 [pg/ml]	0 (0–555.5)	1.5 (0–482.4)	0.25	0.90
TARC/CCL 17 [pg/ml]	304.2 (77.1–4549.4)	391.9 (100.2–2684.6)	0.57	0.35
IgG4 [mg/dL]	45.1 (0–479.0)	22.0 (10.0–332.0)	0.50	0.30
IgG4/IgG ratio*100	6.3 (0–32.0)	3.2 (1.0–29.0)	0.26	0.54
ESR [mm/hour]	5.0 (1–40.0)	8.5 (1–40.0)	0.64	0.074
CRP [mg/L]	2.5 (0.1–32.9)	1.7 (0.1–12.3)	0.74	0.49
Eosinophils (%)	4.0 (0.1–50.0)	4.3 (0.8–33.6)	0.86	0.86

Data are shown as median (range in parenthesis); EGPA: eosinophilic granulomatosis with polyangiitis (Churg-Strauss); LR, Logistic regression analysis as described in Materials and Methods; MWU, Mann Whitney U test; n, number of visits

Next, we asked whether biomarker levels differed between groups defined by use of prednisone or immunosuppressive agents. Results are detailed in **Table 3 and Table 4**. As expected, eosinophil counts were higher in non-users of prednisone than in users, but ESR, CRP, serum TARC/CCL17 and IgG4/IgG ratio was not significantly different. Eotaxin-3 levels were also increased in patients not on prednisone. IgG4 levels were lower in patients not taking prednisone according to unadjusted analysis; however, the difference was not significant after correction for disease activity and use of immunosuppressive agents. Similarly, we observed lower eotaxin-3 levels and a higher ESR in patients on immunosuppressive agents in unadjusted analysis, whereas in the multivariate model, the difference was not significant.

**Table 3:** Serum biomarkers of patients with EGPA based on prednisone usage at time of study visit.

Serum parameters	No pred. use (n = 24)	Pred. use (n = 81)	MWU	LR
Eotaxin-3 [pg/ml]	9.7 (0–549.8)	0 (0–555.5)	**0.004**	**0.021**
TARC/CCL17 [pg/ml]	393.6 (129.3–1918.0)	285.5 (77.1–4549.4)	0.18	0.67
IgG4 [mg/dL]	21.5 (7.0–281.0)	47.0 (7.0–479.0)	0.038	0.099
IgG4/IgG ratio*100	3.4 (0.1–22.0)	6.0 (1.0–32.0)	0.52	0.19
ESR [mm/1st hour]	5.0 (1–40.0)	5.0 (1–40)	0.80	0.084
CRP [mg/L]	2.9 (0.1–32.9)	2.2 (0.1–21.0)	0.41	0.20
Eosinophils (%)	8.0 (1–50.0)	4.0 (0.1–43.0)	**<0.001**	**0.006**

Data are shown as median (range in parenthesis); EGPA: eosinophilic granulomatosis with polyangiitis (Churg-Strauss); LR, Logistic regression analysis as described in Materials and Methods; MWU, Mann Whitney U test; n, number of visits; pred., prednisone

**Table 4:** Serum biomarkers of patients with EGPA based on use of immunosuppressive agents at time of study visit.

Serum parameters	No immunosuppr. use (n = 32)	Immunosuppr. use (n = 73)	MWU	LR
Eotaxin-3 [pg/ml]	1.6 (0–555.5)	0 (0–92.2)	0.015	0.06
TARC/CCL17 [pg/ml]	386.3 (97.1–2684.6)	288.3 (98.8–4549.4)	0.28	0.47
IgG4 [mg/dL]	19.5 (8–479)	62.4 (7–337)	0.15	0.74
IgG4/IgG ratio*100	2.9 (1–32.0)	7.1 (1–28.0)	0.079	0.58
ESR [mm/1st hour]	4 (1–20)	8 (1–40)	0.031	0.07
CRP [mg/L]	3.5 (0.1–21.0)	2.0 (0.1–32.9)	0.23	0.42
Eosinophils (%)	5.5 (0.7–33.6)	4.0 (0.1–50.0)	0.46	0.41

Data are shown as median (range in parenthesis); EGPA: eosinophilic granulomatosis with polyangiitis (Churg-Strauss); immunosuppr., immunosuppressive agents (9 patients were on methotrexate, 7 on azathioprine and 1 on mycophenolate mofetil); LR, Logistic regression analysis as described in Materials and Methods; MWU, Mann Whitney U test; n, number of visits

## Discussion

Little is known about serologic markers of activity in relapsing patients with EGPA. Defining active disease in EGPA is especially challenging because almost all patients have underlying inflammatory disease that does not necessarily indicate activity of EGPA per se, such as asthma and symptoms of rhinosinusitis. Symptoms of asthma may recur together with signs of vasculitis in patients with active EGPA, a worsening of asthma alone, however, is usually insufficient to diagnose a relapse of EGPA.

Many patients are prescribed chronic low dose of prednisone to help with a control of asthma rather than to treat vasculitis or parenchymal eosinophilic inflammation. When both upper and lower respiratory symptoms recur upon prednisone tapering, it is often difficult to decide whether vasculitis itself is active. Blood eosinophil counts are reduced by even low doses of glucocorticoids and are therefore also difficult to interpret in clinical practice. Conversely, elevated eosinophil counts may occur without return of vasculitis.

We studied a set of potential biomarkers (Eotaxin-3, TARC/CCL17, IgG4, and IgG4/IgG ratio) for disease relapse in a prospective, well-defined EGPA cohort. All biomarkers have been previously identified in newly- diagnosed patients with EGPA. TARC/CCL17, a chemokine responsible for Th2 cell recruitment, is elevated in the serum of active patients with EGPA correlating with disease activity [8]. Further, eotaxin-3 is an eosinophil-attracting chemokine elevated in EGPA but not in other eosinophilic or vasculitic disorders [14]. IgG4 is the least abundant IgG subclass. The function of IgG4 is currently unclear but it is associated with a Th2 immune response [9]. IgG4 is highly elevated in active EGPA at disease presentation and decreases during remission. However, prior studies of IgG4 in EGPA included patients who were newly diagnosed and most patients were not on immunosuppressive therapy at the time of testing. Therefore, we studied a real-life cohort of established patients with EGPA focusing on relapsing disease.

In this cohort, most disease flares were mild in the observational period and we could not find any significant differences in routine serologic markers such as ESR, CRP, or eosinophil count. These findings have been corroborated in a more detailed analysis of the same VCRC cohort using data from 141 patients with EGPA with longitudinal follow-up [15]. Organ manifestations of relapsing patients were, however, typical for EGPA with a predominance of constitutional and respiratory symptoms, ENT manifestations and neuropathic pain. Levels of eotaxin-3, TARC/CCL17, IgG4, and IgG4/IgG ratio all failed to discriminate reliably between active and inactive patients. Prednisone clearly modulated serum levels of eotaxin-3 and eosinophils independent of disease activity. Overall, the frequent use of prednisone in EGPA argues against a significant role for these biomarkers in follow-up clinical care. Whether immunosuppressive agents also influence levels of the measured biomarkers is unclear. In our cohort, no statistically significant association was observed in the multivariate analysis; however, the number of patients on immunosuppressive agents was small and several agents with potentially different impact on biomarkers were used. Larger studies are required to investigate the possible association between immunosuppressive drugs and biomarkers in EGPA.

Our study has some important strengths. The study subjects were part of a standardized, prospective, longitudinal data collection with an extensive dataset. In addition, all patients were recruited in expert centers and assessed by clinicians familiar with this rare disease and its clinical manifestations.

Our study has also some limitations: The sample size of our cohort was small compared to biomarker studies in other rheumatic diseases. However, given the rarity of EGPA, it is challenging to assemble larger cohorts for such a project. Most relapses were mild lacking serious clinical manifestations (such as cardiovascular, gastrointestinal and central nervous system involvement), and we cannot exclude the possibility that some patients had their prednisone dose increased before serum sampling. Yet, both of these facts reflect the clinical reality of the disease and its management. Moreover, most patients were already on glucocorticoids at the initiation of study, and detailed information about doses of prednisone and immunosuppressive agents used prior to enrollment were limited, making the analysis of associations between biomarkers and these factors difficult.

In summary, defining biomarkers for relapsing disease in EGPA remains difficult. Novel biomarkers tested in this study could not reliably discriminate active and inactive disease. The potential impact of glucocorticoids and other immunosuppressive drugs with available and potential biomarkers remains a major challenge in the study of biomarkers for EGPA.
